# Relationship between impaired cerebral lymphatic function and iron deposition, blood flow in VaD: a clinical MRI study

**DOI:** 10.3389/fnins.2025.1666038

**Published:** 2025-12-03

**Authors:** Yingqi Lu, Yushuang Liu, Haoran Zhang, Huixing Wu, Yuan Long, Cong Pang, Juncheng Xi, Zhongling Zhang, Jinping Xu, Shangjie Chen

**Affiliations:** 1 Department of Rehabilitation Medicine, The People’s Hospital of Baoan Shenzhen, Shenzhen, China; 2 Institute of Biomedical and Health Engineering, Shenzhen Institutes of Advanced Technology, Chinese Academy of Sciences, Shenzhen, China; 3 Guangzhou Geriatric Hospital, Guangzhou, China; 4 Department of Neurology, The First Affiliated Hospital of Harbin Medical University, Harbin, China

**Keywords:** vascular dementia (VaD), quantitative susceptibility mapping (QSM), arterial spin labeling (ASL), diffusion tensor imaging (DTI), cerebral blood flow (CBF), diffusion tensor image analysis along the perivascular space (DTI-ALPS)

## Abstract

**Background:**

The neural mechanisms induced by cerebral small vessel disease (CSVD) in the vascular dementia (VaD) is extremely complex. Recent studies have identified altered lymphatic function as a key factor contributing to the development of cognitive deficits, whether they linked to iron deposition and reduced cerebral blood flow remains unclear.

**Methods:**

The study involved 59 participants, comprising 30 healthy controls and 29 patients with VaD. Each participant underwent QSM, ASL, and DTI imaging scans and clinical measurements. The ALPS index based on DTI, QSM, and ASL for the left and right hemispheres, as well as for the whole brain were calculated and compared between groups. Regional mean susceptibility and mean CBF based on AAL template were calculated and compared between groups. Correlation analyses were performed between altered indexes and clinical measurements. Finally, mediation analyses were conducted to determine whether the mean susceptibility rate and mean CBF were involved in the modulation process of the ALPS.

**Results:**

DTI-ALPS based analysis demonstrated a significantly lower ALPS index for the left, right, and whole brain in the VaD as compared to healthy controls. Mean susceptibility in the bilateral superior occipital gyrus and bilateral middle occipital gyrus was significantly higher, whereas mean CBF in the eft inferior frontal gyrus, opercular part, right rolandic operculum, right insula, and right heschl gyrus was significantly lower in the VaD. Further correlation analyses revealed associations between elevated mean susceptibility, reduced ALPS index, and cognitive impairments. Additionally, the results of correlation and mediation analyses suggested that CBF may be involved in the regulatory process of DTI-ALPS.

**Conclusion:**

Our results demonstrated that altered lymphatic function is a key pathological factor in VaD, and reduced cerebral blood flow may play a role in this process. These findings provide a theoretical basis for identifying new targets in the treatment of VaD.

## 1 Introduction

Cerebral small vessel disease (CSVD) is commonly observed in the elderly and is strongly associated with conditions such as hypertension, diabetes mellitus, and aging. It presents a range of clinical signs, such as cognitive deficits, movement abnormalities, and emotional disorders, which significantly impact the quality of life of patients and contribute to the societal burden (Hainsworth et al., 2024). In recent years, with advancements in imaging technology, significant progress has been made in understanding the diagnosis and neural mechanisms associated with CSVD. Imaging techniques, such as magnetic resonance imaging (MRI), can detect characteristic manifestations of CSVD, including cerebral white matter hyperintensities, lacunar cerebral infarctions, and cerebral microhemorrhages (Goeldlin et al., 2022).

However, the relationship between vascular dementia (VaD) and CSVD is complex and multifaceted, involving the interplay of various vascular, neurological, and systemic factors. This topic has garnered significant attention in recent research. The newest neuroimaging studies reveal that patients suffering from VaD experience numerous changes in brain structure and function (Folloso et al., 2024). Resting-state magnetic resonance imaging (fMRI) studies of brain function have revealed significant decreases in brain network characteristics in patients with VaD, which are correlated with cognitive decline (Wang et al., 2024). Atrophy of hippocampal volume, posterior thalamic radiation, cingulate gyrus, and other multiple sites is hallmark signs of precursor damage in VaD (Manco et al., 2024). Hence, examining the modified neural pathways of VaD is of great significance, as it can offer a foundation for diagnosis and inform targeted therapies in medical practice.

The diffusion tensor imaging analysis along the perivascular space (DTI-ALPS; ALPS index) is a non-invasive method for assessing cerebrospinal fluid alterations (Li et al., 2024). Since its proposal in 2017, this technique has been gradually adopted for the observation of different neurodegenerative diseases (Taoka et al., 2017). Previous studies have found that a reduced ALPS index is associated with executive dysfunction in patients with CSVD (Liu et al., 2025). In another study, researchers analyzed the correlation between the ALPS index and cognitive function in 133 Alzheimer’s disease (AD) patients. They found that ALPS index mediated the relationship between white matter hyperintensity load and cognitive function (Hong et al., 2024). Quantitative susceptibility mapping (QSM) leverages the differences in the magnetization properties of various substances under a magnetic field (Merenstein et al., 2025). It can be used to obtain a map of the susceptibility distribution in local tissues by removing background field effects, performing inverse calculations, and other processing steps (Jang et al., 2025). QSM-based analyses are increasingly gaining acceptance and are beginning to be widely used in research. Iron deposition at multiple sites, including the basal ganglia, superior frontal gyrus, and substantia nigra, is strongly associated with the development of cognitive impairment (Li et al., 2022; Tu et al., 2022; Luyken et al., 2025). In our earlier research, we discovered iron accumulation in several areas, such as the caudate nucleus, cerebellum, frontal lobe, and paracentral lobule, during the early stages of VaD, which was linked to cognitive deterioration (Liu et al., 2025). Cerebral blood flow (CBF) is controlled by intricate physiological processes that are affected by a variety of neural, metabolic, and humoral elements (Fatima et al., 2025). There are several methods for measuring CBF, including arterial spin labeling (ASL), positron emission tomography (PET), and single photon emission computed tomography (SPECT) (Padrela et al., 2025; Ibaraki et al., 2025). ASL, for example, measures CBF non-invasively by labeling water molecules in arterial blood. It offers the advantages of being relatively easy to perform and free from radiation exposure (Chen et al., 2024). Studies have shown that in the assessment of cerebral ischemic disease, CBF measured by ASL are in good agreement with conventional PET measurements, providing a reliable basis for clinical diagnosis (Baskerville et al., 2012). At the same time, the decline in CBF is considered a risk factor for cognitive dysfunction in patients with AD and VaD (Chen et al., 2025). Several relevant studies have explored the use of ALPS index, QSM, and CBF for VaD induced by CSVD. However, to date, no study has simultaneously described modality-specific changes in ALPS index, QSM, and CBF in VaD patients, and the intrinsic associations among these three factors remain unclear. Therefore, studies that combine ALPS index, QSM, and CBF may offer new insights into the neural mechanisms underlying the disease.

In this study, to further explore the intrinsic neural mechanisms of VaD, we hypothesize that changes in lymphatic function are crucial in the cognitive decline seen in VaD patients, with iron buildup and decreased cerebral blood flow influencing lymphatic activity and linked to cognitive decline. To test this hypothesis, we collected DTI, QSM, and ASL images from participants at two different stages of HC and VaD. The ALPS index was explored through DTI-ALPS analysis in relation to cognitive function alterations. CBF were obtained from ASL images, and susceptibility were derived from QSM images. The mean CBF and mean susceptibility of regions of interest (ROIs) were calculated based on the anatomical auto-labeling (AAL) template. CBF and susceptibility for the left, right, and whole brain were calculated separately, and their association with the ALPS index was probabilistically examined. Finally, we assessed how well the observed brain changes were associated with cognitive function.

## 2 Materials and methods

### 2.1 Participates

The Institutional Review Board of Guangzhou Geriatric Hospital approved this study (Approval No. LW-Z20221125), with all participants signing informed consent forms prior to their involvement.

Healthy controls (HC) were required to: (1) Be aged 55 to 85 years, of either gender; (2) Be right-handed; (3) Show no significant decline in memory or cognitive abilities; (4) Montreal Cognitive Assessment (MoCA) score ≥ 26, Mini-Mental State Examination (MMSE) score ≥ 27; (5) No reasons to avoid magnetic resonance imaging; and (6) Informed consent form signed voluntarily.

Participants in the VaD were required to: (1) Cranial MRI showing cerebrovascular disease, classified as Fazekas grade 1–2.; (2) Memory loss reported by the individual or confirmed by an informant, with at least one link between the start or worsening of symptoms and the beginning of cerebrovascular disease, or a Hachinski Ischemia Scale (HIS) score ≥ 7; (3) MoCA score < 26, MMSE score ≤20 for individuals with a primary school education and ≤ 24 for those with secondary school education or higher (illiterate individuals could not participate); (4) Ability to cooperate in completing the study; and (5) The participants and their families provided informed consent and voluntarily signed the consent form.

The exclusion criteria were as follows: (1) Patients diagnosed with other types of dementia; (2) Individuals who do not cooperate with the treatment or refuse to sign the informed consent form; (3) Patients with severe neurological deficits, including aphasia, visual and auditory disorders, or significant hand hemiparesis; (4) Patients with coexisting psychiatric disorders, such as severe anxiety, depression, or schizophrenia; and (5) Individuals in the acute phase of significant cerebral infarction, brain bleeding, or subarachnoid hemorrhage, or those with a history of cerebrovascular abnormalities or aneurysmal subarachnoid hemorrhage, or who possess an untreated aneurysm (diameter >3).

The trial included a total of 59 participants, comprising 30 HC and 29 individuals with VaD.

### 2.2 MRI data acquisition

The Siemens 3.0 T MAGNETOM Lumina MRI scanner was utilized for the participants. Each participant underwent comprehensive brain imaging, which included 3D T1, FLAIR, QSM, ASL, and DTI, with detailed scanning parameters provided below.

3D T1: The repetition time (TR) = 2,200 ms; echo time (TE) = 2.43 ms; field of view (FOV) = 256 mm × 256 mm; slice thickness = 1.0 mm; distance factor = 0.5 mm; flip angle (FA) = 8°; number of slices = 176; and voxel size = 1.0 mm × 1.0 mm × 1.0 mm.
FLAIR: TR = 8,000 ms; TE = 89 ms; acquisition matrix = 185 × 304; FOV = 230 mm × 199.7 mm; slice thickness = 5.0 mm; slice gap = 1 mm; FA = 120°; and total number of slices = 20 axial images.
QSM: TE = 4.64 ms, 7.04 ms, 9.77 ms, 12.50 ms, and 16.25 ms. FA = 15°; acquisition matrix = 193 × 256; slice thickness = 2.0 mm; total number of slices = 80; and voxel size = 0.43 mm × 0.43 mm × 2.0 mm.
ASL: The perfusion mode is PCASL; TR = 5,600 ms; TE = 20.3 ms; FA = 180°; acquisition matrix = 62 × 64; slice thickness = 3.0 mm; total number of slices = 40; voxel size = 1.5 mm × 1.5 mm × 3.0 mm.
DTI: TR = 8,700 ms; TE = 92 ms; acquisition matrix = 128 × 128; slice thickness = 2.0 mm; total number of slices = 76; voxel size = 2.0 mm × 2.0 mm × 2.0 mm; including 20 diffusion gradient directions (1 b = 0 s/mm^2^ and 60 b = 1,000 s/mm^2^).

### 2.3 Total SVD score

The total SVD score was assessed based on FLAIR imaging to reflect the overall imaging burden of CSVD (Nguyen et al., 2025). We also recorded the total SVD score for each participant, reflecting the overall imaging burden of cerebral small-vessel disease. The score ranges from 0 to 4, with one point assigned for each of the following: (1) extensive white-matter hyperintensities (Fazekas ≥ 2); (2) at least one lacunar infarct; (3) enlarged perivascular spaces in the basal ganglia (score ≥ 2); and (4) at least one cerebral microbleed. Higher scores indicate a greater CSVD imaging load.

### 2.4 DTI-ALPS analysis

The DTI data were initially pre-processed using FSL,^1^ which included denoising, head motion correction, and eddy current correction. An anisotropy fraction (FA) map was also created during pre-processing, along with direction maps for the x-, y-, and z-axes. Following this, we generated four spherical regions of interest, each having a diameter of 5 mm, using the equation below:


$$\text{ALPS index}=\frac{\text{mean}\;(\text{Dxxproj},\text{Dxxassoci})}{\text{mean}\;(\text{Dyyproj},\text{Dzzassoci})}$$

The ALPS values were determined by calculating the ratio of two sets of diffusivity values that are perpendicular to primary fibers in tissue. In the equation, Dxxproj, Dxxassoci, Dyyproj and Dzzassoci represent the x-axis diffusion coefficient of projected fiber region, the x-axis diffusion coefficient of associated fiber region, the y-axis diffusion coefficient of projected fiber region, and the z-axis diffusion coefficient of associated fiber region, respectively. And we also calculated the mean ALPS for the left, right, and whole brain.

### 2.5 Susceptibility analysis

The pre-processing and reconstruction of the QSM data were primarily performed using the SEPIA software (One-stop QSM processing)^2^ and SPM12. The specific steps included pre-processing the phase and amplitude maps of original QSM using SEPIA, followed by reconstruction of QSM map through brain extraction, phase unfolding, and background field elimination. Next, we used SPM12 to align reconstructed QSM image with T1 image. The T1 image was normalized to MNI space, and the QSM image was normalized using deformation field. Subsequently, we smoothed the normalized QSM images using a 6 × 6 × 6 mm^3^ Full-Width Half-Height (FWHM) Gaussian kernel. Finally, we extracted the mean susceptibility for the corresponding brain regions of QSM images based on AAL90 template. Additionally, we calculated the mean susceptibility for the left, right, and whole brain, respectively.

### 2.6 ASL analysis

First, we extracted cerebral blood flow (CBF) values based on calculations from pCASL sequences as described in previous studies (Wang et al., 2008; Raoult et al., 2011). The specific formula is as follows:


$$\mathrm{CBF}=\frac{6000\cdot \lambda \cdot ({\mathrm{SI}}_{\text{control}}-{\mathrm{SI}}_{\text{label}})\cdot e^{\frac{\mathrm{TI}}{T_{1},\text{blood}}}}{2\cdot \alpha \cdot {\mathrm{TI}}_{1}}[\mathrm{ml}/100\;g/\min]$$

**Table:** 

Parameter	Value
λ (blood–brain partition coefficient)	0.9mL/g
$T_{1,\text{blood}}$ at 3.0 T	1650ms
α (labeling efficiency) for PCASL	0.85

Next, we used SPM12 to align reconstructed CBF image with T1 image. The T1 image was normalized to MNI space, and the CBF image was normalized using deformation field. We smoothed the normalized CBF images using a 6 × 6 × 6 mm^3^ FWHM Gaussian kernel. Finally, we extracted the mean CBF for the corresponding brain regions of CBF images based on AAL90 template. Additionally, we calculated the mean CBF for the left, right, and whole brain, respectively.

### 2.7 Statistical analysis

The data was statistically analyzed using IBM SPSS 26.0, and a *p* < 0.05 was deemed to consider a significant difference. Continuous data are presented as mean ± standard deviation. A two-sample *t*-test was used to compare differences in age, a chi-square test was employed to compare gender differences, and a Kruskal-Wallis test was used to compare clinical data between the two groups. Moreover, the data for QSM, CBF, and ALPS index were compared through general linear modeling (GLM), including gender and age as covariates. Partial correlation analyses were used to evaluate the relationships between the three modalities and their connections with clinical data, with results adjusted for false discovery rate (FDR) multiple comparisons. Finally, the mediating relationship between the ALPS index and clinical data was examined through mediation analysis.

## 3 Results

### 3.1 Demographic and clinical characteristics

This study involved 59 participants, including 30 HC and 29 individuals diagnosed with VaD. The demographic and clinical characteristics of participants are presented in Table 1. The mean age of HC group was 78.13 ± 6.11 years, consisting of 10 males and 20 females. And in VaD group, the mean age was 80.66 ± 9.56, consisting of 13 males and 16 females. There were no significant differences in age and gender between the two groups (*p* > 0.05). Conversely, the clinical scales of MMSE, MoCA, and Total SVD Score showed significant differences between the two groups (*p* < 0.001).

**Table 1:** Demographic and clinical data for all participants.

Groups	HC	VaD	t/χ^2^/z value	*p* value
Subjects	30	29	–	–
Age (mean ± SD)	78.13 ± 6.11	80.66 ± 9.56	8.708	0.165 ^a^
Sex (male/female)	10/20	13/16	–	0.052 ^b^
MMSE (mean ± SD)	28.93 ± 1.20	22.34 ± 4.27	50.654	<0.001^c^
MoCA (mean ± SD)	27.50 ± 1.22	18.66 ± 4.70	64.715	<0.001 ^c^
Total SVD Score (mean ± SD) (Range 0–4)	2.47 ± 0.86	3.52 ± 0.63	15.473	<0.001 ^c^

a Represents two sample *t* texts.

b Represents chi-square test.

c Represents Kruskal-Wallis test.

HC, healthy controls; VaD, vascular dementia; MMSE, Mini-Mental State Examination; MoCA, Montreal Cognitive Assessment; and SVD, small vessel disease.

### 3.2 DTI-ALPS analysis

**Figure 1:**
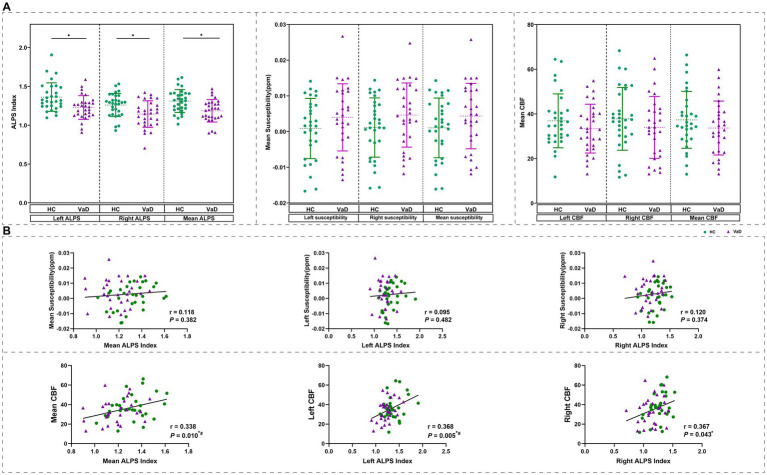
Change in ALPS index based on DTI-ALPS analysis. **(A)** The mean ALPS index was different between two groups obtained by GLM with age, gender as covariates. And the difference of mean susceptibility and mean CBF were obtained by GLM with age, gender as covariates. **(B)** Results of correlation analyses with age, gender as covariates between mean QSM\mean CBF, and mean ALPS index in all participants. * Represents *p* < 0.05, # represents FDR *p* < 0.05. HC, healthy controls; VaD, vascular dementia; CBF, cerebral blood flow; and ALPS, diffusion tensor image analysis along the perivascular space.

Next, we investigated changes in the ALPS index in patients with VaD. We derived the mean ALPS index for the entire brain and also for the left and right cerebral hemispheres. Comparison using GLM, with gender and age as covariates, revealed a trend of decreased mean ALPS index as well as left and right ALPS index in VaD compared to HC (*p* < 0.05). In addition, we calculated the mean susceptibility for whole brain, as well as for the left and right cerebral hemispheres in both HC and VaD groups. Similarly, we computed the mean CBF for whole brain and the left and right cerebral hemispheres, based on the values extracted from AAL template. Statistical analysis revealed that although there was a trend towards increased susceptibility and decreased CBF in VaD compared to HC, no significant differences were observed (*p* > 0.05) (Figure 1A). Furthermore, we conducted a correlation analysis involving the ALPS index and the mean susceptibility/ CBF, considering gender and age as covariates. The results showed that the whole brain mean ALPS index was significantly correlated with the whole brain mean CBF (*r* = 0.338, *p* = 0.010). The mean ALPS index on the left side was significantly correlated with the mean CBF on the left side (*r* = 0.368, *p* = 0.005), and the mean ALPS index on the right side was significantly correlated with the mean CBF on the right side (*r* = 0.367, *p* = 0.043). In contrast, no significant correlation was found between ALPS and susceptibility values (*p* > 0.05) (Figure 1B).

### 3.3 Susceptibility analysis

**Figure 2:**
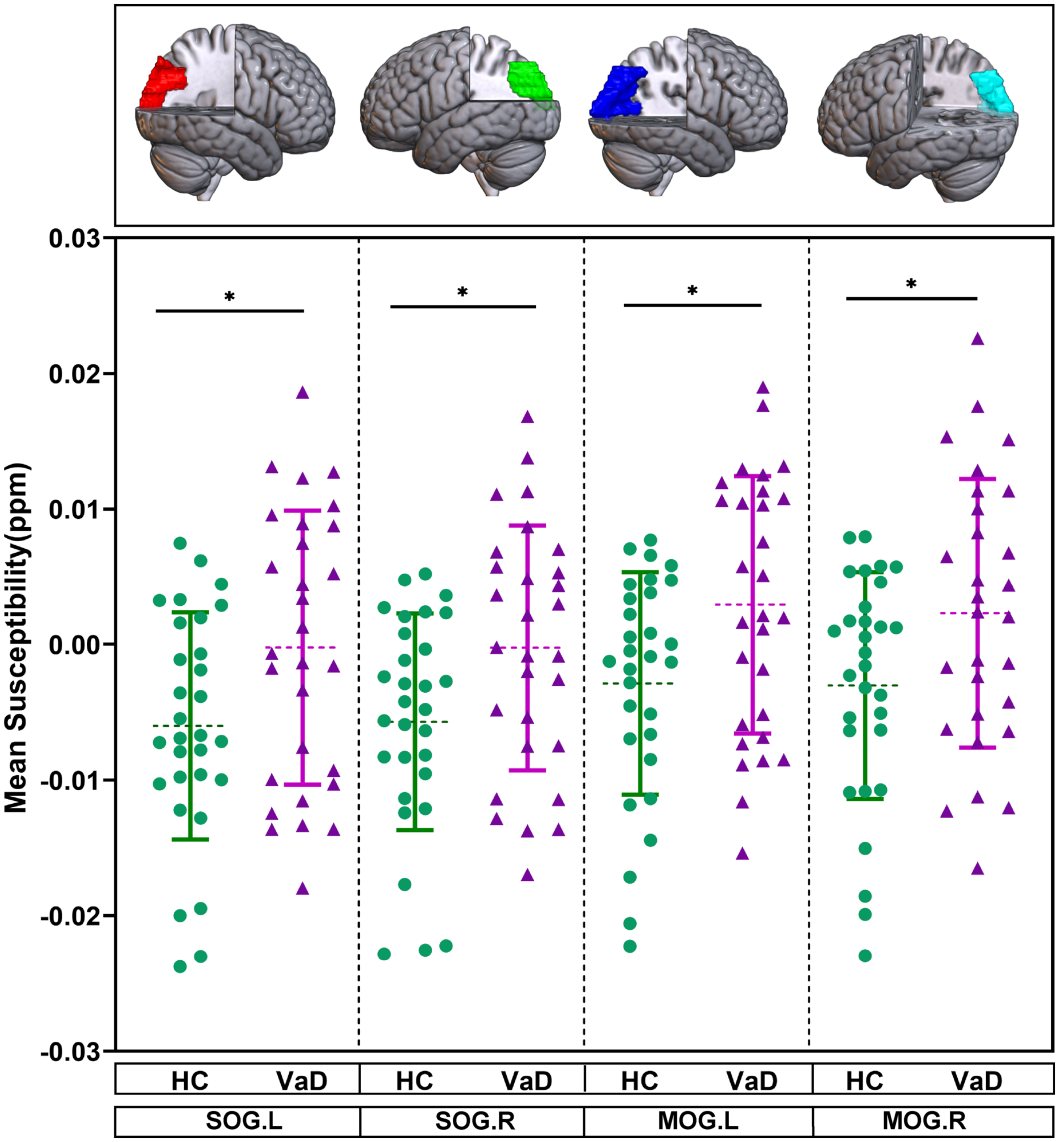
Brain regions showing altered mean susceptibility in the ROI-based analysis. They were obtained by GLM with age, gender as covariates. * Represents *p* < 0.05. HC, healthy controls; VaD, vascular dementia; SOG.L, left superior occipital gyrus; SOG.R, right superior occipital gyrus; MOG.L, left middle occipital gyrus; and MOG.R, right middle occipital gyrus.

The mean susceptibility of 90 brain regions were compared between HC and VaD by using GLM, with gender and age as covariates. It was found that the mean susceptibility of bilateral superior occipital gyrus (SOG.L, SOG.R) and bilateral middle occipital gyrus (MOG.L, MOG.R) tended to be higher in VaD compared to HC (*p* < 0.05) (Figure 2).

### 3.4 ASL analysis

**Figure 3:**
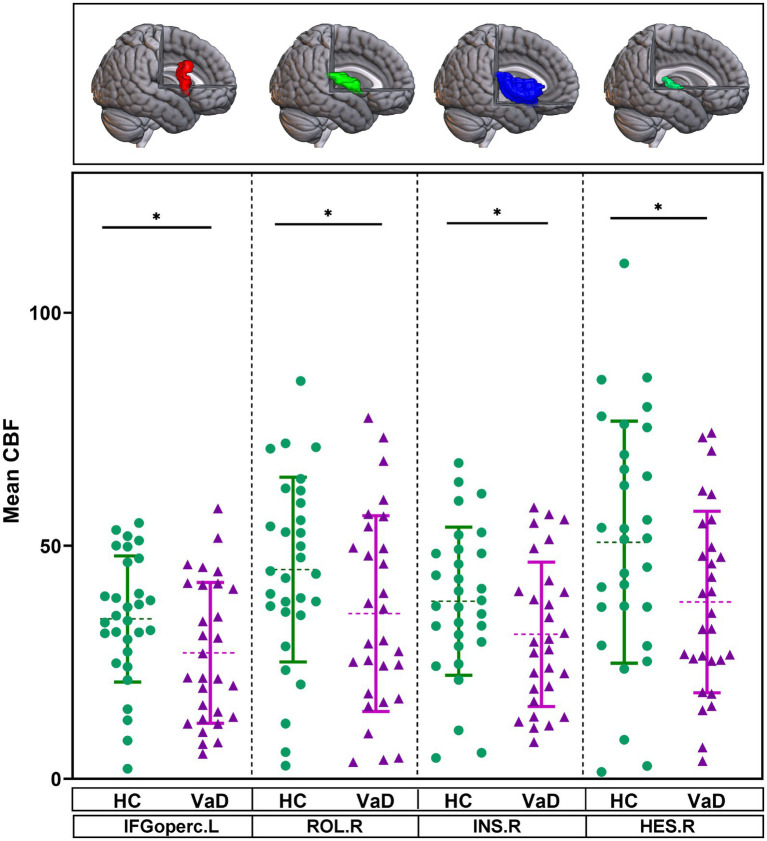
Brain regions showing altered mean CBF in the ROI-based analysis. They were obtained by GLM with age, gender as covariates. * Represents *p* < 0.05. HC, healthy controls; VaD, vascular dementia; CBF, cerebral blood flow; IFGoperc.L, left inferior frontal gyrus, opercular part; ROL.R, right rolandic operculum; INS.R, right insula; and HES.R, right heschl gyrus.

The mean CBF of 90 brain regions were compared between HC and VaD by using GLM, with gender and age as covariates. In VaD patients, it was observed that the mean CBF in four brain regions—the left inferior frontal gyrus, opercular part (IFGoperc.L), right rolandic operculum (ROL.R), right insula (INS.R), and right heschl gyrus (HES.R)—tended to decrease compared to HC (*p* < 0.05) (Figure 3).

### 3.5 Associations among imaging indexes and clinical data

**Figure 4:**
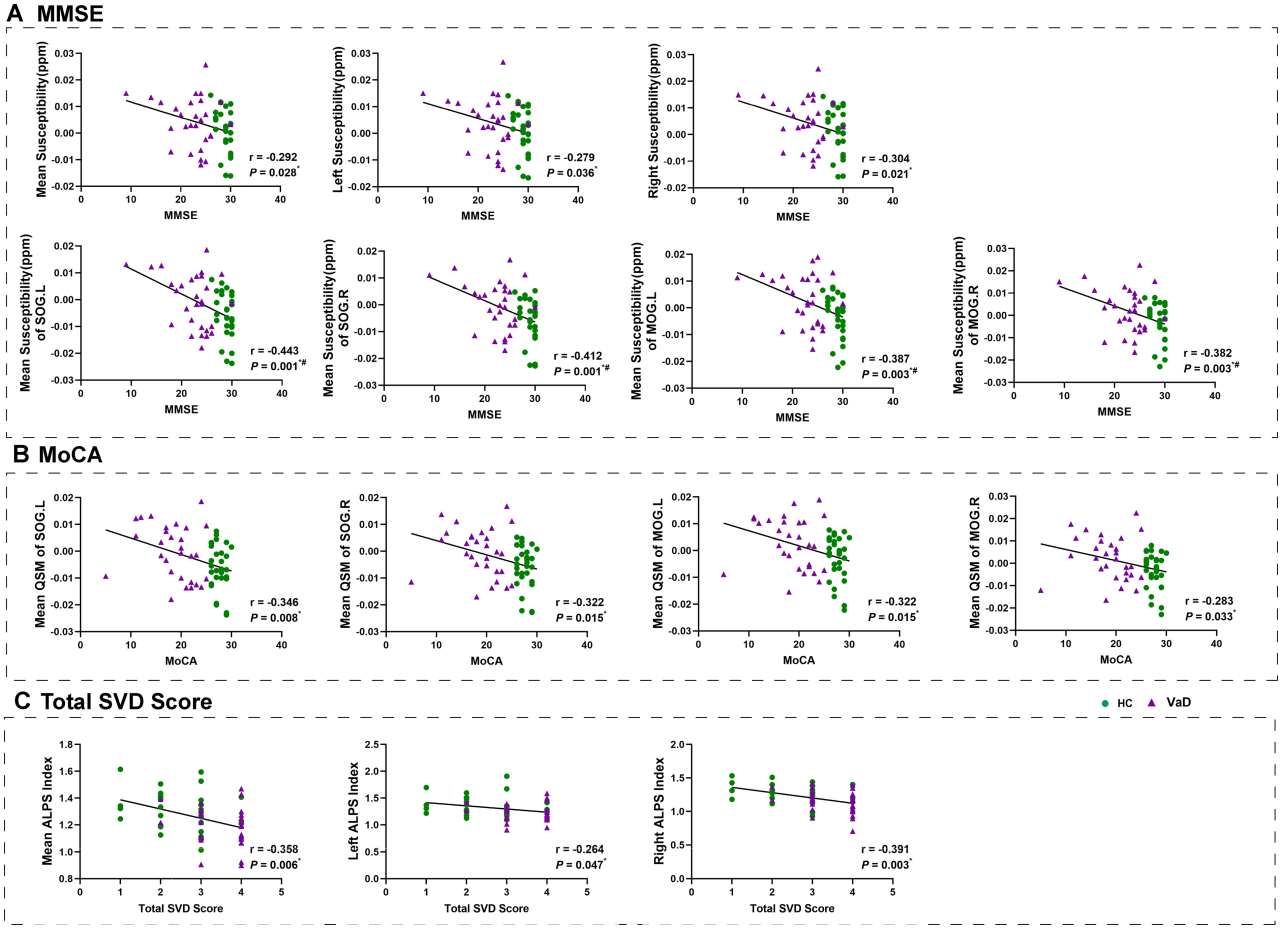
Results of correlation analyses with age, gender as covariates between clinical measurements and mean susceptibility, ALPS index in all participants. * Represents *p* < 0.05, # represents FDR *p* < 0.05. HC, healthy controls; VaD, vascular dementia; SOG.L, left superior occipital gyrus; SOG.R, right superior occipital gyrus; MOG.L, left middle occipital gyrus; MOG.R, right middle occipital gyrus; ALPS, diffusion tensor image analysis along the perivascular space; MMSE, Mini-Mental State Examination; MoCA, Montreal Cognitive Assessment; and SVD, small vessel disease.

After adjusting for gender and age in the whole group, we found that higher susceptibility was associated with lower MMSE and MoCA scores. We also conducted a correlation analysis of the mean susceptibility of bilateral superior occipital gyrus and bilateral middle occipital gyrus, after zoning, with the MMSE and MoCA scores. The results were consistent with the current analysis (Figures 4A,B). In addition, the whole-brain ALPS index, left ALPS index, and right ALPS index were found to be associated with lower total SVD scores (Figure 4C).

### 3.6 Mediation analysis

**Figure 5:**
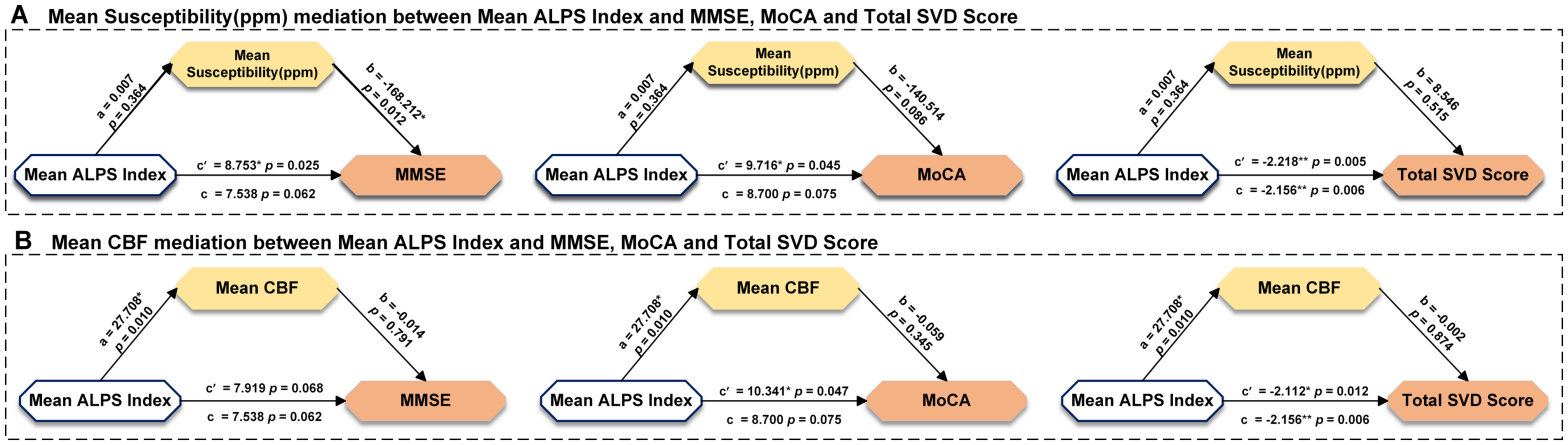
Results of mediation analysis with age, gender as covariates among mean susceptibility, mean CBF, ALPS index, and clinical measurements in all participants. * Represents *p* < 0.05, ** represents *p* < 0.001, c represents direct effect, c’ represents indirect effect. MMSE, Mini-Mental State Examination; MoCA, Montreal Cognitive Assessment; CBF, cerebral blood flow; and ALPS, diffusion tensor image analysis along the perivascular space.

We further investigated whether the mean ALPS index in VaD patients mediated cognitive decline using a mediation model. We set the mean ALPS index as the independent variable, the mean susceptibility as the mediator variable, and MMSE, MoCA, and Total SVD score as the dependent variables, respectively, with gender and age included as covariates. It was found that the mean ALPS index was directly correlated with the Total SVD score, but the mean susceptibility was not involved in the association between the mean ALPS index and MMSE, MoCA, or Total SVD score (Figure 5A). Then, we set the mean ALPS index as the independent variable, mean CBF as the mediator variable, and MMSE, MoCA, and Total SVD score as the dependent variables, with gender and age included as covariates. It was found that the indirect predictive effect of the mean ALPS index on MMSE, MoCA, and Total SVD score was not significant when mean CBF was the mediating variable (Figure 5B).

## 4 Discussion

We identified that the ALPS index was significantly lower in VaD patients and correlated with the Total SVD score. The global mean ALPS index was significantly correlated with the global mean CBF, but not with the global mean susceptibility. Additionally, VaD patients exhibited significantly higher susceptibility in the bilateral superior occipital gyrus and bilateral middle occipital gyrus compared to HC, and these changes were correlated with MMSE and MoCA scores. CBF was significantly reduced in four brain regions—IFGoperc.L, ROL.R, INS.R, and HES.R—in VaD patients.

This study included observations based on DTI-ALPS. The lymphatic system helps process brain waste products, including Aβ and tau proteins, and some researchers believe that dysfunction in this system may occur before the onset of cognitive impairment (Kamagata et al., 2022). In VaD, small-vessel disease is a primary causative factor and is closely linked to normal or impaired lymphatic function (Hong et al., 2024). We also examined the changes in ALPS index in both right and left cerebral hemispheres and found an absence of bilateral symmetry. This might suggest that vascular factors and nearby structures could affect the differences between the left and right hemispheres. The correlation analysis with clinical scales revealed a significant relationship between the ALPS index and the total SVD score. The total SVD score, which combines multiple imaging markers, offers a more comprehensive assessment of the overall burden of CSVD and plays a crucial role in predicting VaD (Siddiquee et al., 2025; Kitagawa et al., 2024). Our results indicate that brains affected by VaD may experience more widespread and severe impairment of lymphatic system function, which suggest that the lymphatic system function may be more extensively and severely impaired in brains affected by VaD.

Additionally, since ALPS index was calculated for the left and right hemispheres as well as the whole brain, we also computed the mean susceptibility and mean CBF for the left side, right side, and entire brain based on the AAL template. These values were then correlated with the ALPS index to examine their relationship. We found that the ALPS index was not significantly correlated with QSM, but there was a significant correlation with CBF. The underlying cause may be that white matter regions are supplied by perforating arteries that branch from the major brain vessels, making them sensitive to ischemic injury (Cai et al., 2022; Plog and Nedergaard, 2018; Funaki et al., 2018). Inadequate cerebral perfusion can impair lymphatic function, disrupting normal mobility, and together these factors contribute to cognitive impairment. Although there are fewer studies on VaD, we found that this trend aligns with previous observations in conditions involving damage to small cerebral vessels, such as smoky disease. This suggests that the co-variation between the ALPS index and CBF could be a potential risk factor for CSVD (Jin et al., 2024).

In contrast to HC, our results revealed iron deposition in bilateral superior occipital gyrus and bilateral middle occipital gyrus of VaD patients, which was correlated with cognitive dysfunction. The occipital lobe is a crucial region of the brain associated with visual processing and spatial cognition. It plays a key role in spatial cognition and memory encoding by integrating visual information processing and cross-modal integration (Lugtmeijer et al., 2025). Patients with VaD experience inefficient visual processing and are unable to effectively integrate visuospatial information, which can impair their ability to perceive and respond to spatial cues (Yoshida et al., 2025). Studies have shown that structural damage to the occipital lobe and lingual gyrus occurs in the early stages of CSVD (Liu et al., 2020). In addition, the occipital lobe communicates information to other areas of the brain through superior temporal sulcus and parietal area, which in turn supports the processing of higher-level tasks such as movement and language (Dadario et al., 2023). Iron deposition has now become a key pathological marker of CSVD. Researchers believe that during the progression of CSVD, the blood–brain barrier (BBB) becomes compromised, leading to the infiltration of toxic fluids and plasma proteins into healthy blood vessels, which in turn results in brain iron deposition (Wardlaw et al., 2019). For patients with CSVD, heightened susceptibility in subcortical regions such as the caudate nucleus, putamen, and pallidum, as well as in cortical areas like the temporal and parietal lobes, is regarded as a risk factor for cognitive dysfunction (Tu et al., 2022; Gao et al., 2025; Sui et al., 2025). Our study found increased susceptibility in the supraoccipital gyrus and mid-occipital gyrus, which strongly correlated with cognitive decline in patients with VaD caused by CSVD. This result further proves that injury to the BBB causes substantial harm in localized brain areas. Our findings suggest that the iron deposition in the supraoccipital and mid-occipital gyrus may contribute to the dysfunction of visual processing and spatial cognition in VaD patients. This further emphasizes the importance of the occipital lobe in cognitive decline, as it is crucial for integrating visual information. Future studies should explore the role of iron accumulation in these regions over time to better understand its impact on disease progression and cognitive impairment. This study conducted QSM analysis based on the AAL template, including the hippocampus, putamen, and thalamus. Although the analysis of these regions did not yield significant results, this may be due to the small sample size and insufficient statistical power. Future studies will expand the sample size and further evaluate iron deposition and atrophy across the whole brain, exploring their relationship with cognitive function.

Our ASL analysis results showed a significant decrease in CBF in four brain regions—IFGoperc.L, ROL.R, INS.R, and HES.R—in patients with VaD. In a healthy state, CBF supplies essential substances, such as oxygen and glucose, required by the brain to maintain proper function (Wright et al., 2025). However, in disease states, particularly with characteristic CSVD lesions such as white matter hyperintensities and the occurrence of cerebral microinfarcts, endothelial cells and astrocytes in the cerebral blood vessels are damaged, inhibiting vasodilatory function (Wang et al., 2024; Wong et al., 2019). This results in a localized reduction of CBF, placing neurons in a state of energy deprivation (Zhang et al., 2024; Zhang et al., 2022). The IFGoperc.L is a crucial brain area that exhibits abnormalities in individuals with cognitive impairment (Becker et al., 2013). Disruption of the white matter microstructure in this area affects the preservation of working memory and results in cognitive deficits (Yang et al., 2020). A complex network of neural connections exists between rolandic operculum, parahippocampal gyrus, and temporal lobe, which plays a crucial role in integrating sensory and auditory information (Mălîia et al., 2018). Previous studies have also shown that CSVD patients exhibit impaired function in this brain region. Our study adds evidence to the relationship between structural and functional impairments in this area and cognitive difficulties (Wang et al., 2025). The insula is a central hub in the somatosensory network and is closely connected with several brain regions, including the frontal lobe, occipital lobe, and limbic system (Simone et al., 2025). A meta-analysis showed that a decrease in insular gray matter is linked to cognitive impairment in CSVD (Li et al., 2023). The heschl gyrus is a key brain region involved in phonological processing, and dysfunction in this area negatively impacts the maintenance of cognitive performance (Roll, 2024; Tzourio-Mazoyer and Mazoyer, 2017). Our study further shows that, in patients with CSVD-induced VaD, the decline in CBF in certain brain regions, may serve as a potential risk factor for developing cognitive impairment. Although this study found correlations between impaired lymphatic function, cerebral blood flow, and iron deposition with VaD, due to the cross-sectional design, only associations are established, and causal relationships cannot be inferred. Future longitudinal or interventional studies may help further explore the specific roles of these factors in the pathogenesis of VaD. Mediation analyses were conducted to explore the potential mechanistic roles of impaired lymphatic function, CBF, and iron deposition in VaD; however, the indirect effects did not reach statistical significance. Therefore, these results should be interpreted with caution, and causality or mechanistic conclusions should not be inferred. Nevertheless, this study still provides important preliminary evidence for exploring the relationship between impaired lymphatic function, CBF, and iron deposition in VaD. Future research, particularly longitudinal or interventional studies, will help further explore the specific roles of these factors in the pathogenesis of VaD.

Based on the findings of this study, the interactions between impaired lymphatic function, reduced CBF, and iron accumulation open new possibilities for targeted interventions in the treatment of VaD and CSVD. These findings suggest that modulating lymphatic flow and iron homeostasis could be potential therapeutic approaches. Firstly, improving lymphatic flow in the brain may help restore proper waste clearance and reduce the accumulation of harmful substances, which are associated with cognitive decline. Given the role of the lymphatic system in maintaining brain health, future interventions to promote lymphatic function could include physical therapies, medications, or other methods aimed at enhancing cerebrospinal fluid circulation. These approaches may have the potential to slow or even halt the progression of VaD, but further research and clinical trials are needed to assess their feasibility and effectiveness (Thipani Madhu et al., 2024). Secondly, modulating iron homeostasis could offer another therapeutic approach. Iron accumulation in the brain is a well-established marker in both CSVD and VaD, and excess iron can lead to oxidative stress and neuronal damage. Iron chelation therapy or other methods to regulate iron levels in the brain could help reduce iron-induced neurodegeneration, thereby mitigating cognitive impairment (Awad et al., 2025; Boccia et al., 2025). Future clinical trials should explore the safety and efficacy of such interventions, particularly in patients at early stages of VaD, to determine if this approach can delay disease progression. In summary, while these interventions are not yet clinically implemented, the study’s findings suggest that targeting lymphatic flow and iron balance may offer promising strategies for future VaD treatment. Further studies and trials will be crucial to determine the practical application of these interventions and their potential benefits for VaD patients.

We corrected for multiple comparisons using the FDR at *p* < 0.05. Compared with the traditional Bonferroni procedure, FDR offers greater statistical power while controlling the expected proportion of false positives, and is now standard in neuroimaging studies with numerous correlated ROIs (Gossmann et al., 2018). Recent investigations employing QSM, ASL analyses have consistently applied FDR correction (Zhang et al., 2025; Pelizzari et al., 2019), demonstrating that it strikes an appropriate balance between Type I error control and sensitivity. We therefore adopted the same approach to ensure reproducibility and comparability with the current literature.

The study identified changes in multiple brain regions in patients with VaD that evolve as the disease progresses. These changes have the potential to serve as useful clinical biomarkers, especially for early diagnosis and monitoring disease progression. However, integrating these MRI-based biomarkers into routine clinical practice presents several challenges. A key issue is validating the biomarkers’ stability, reliability, and broad applicability in different clinical settings. Additional studies are needed to assess their performance across diverse patient populations, scanning devices, and imaging protocols. Machine learning techniques could be applied to evaluate their consistency in various contexts. While these structural changes show promise in detecting vascular cognitive impairment, their specificity and ability to distinguish between similar conditions, especially in the early stages, require further validation. At this stage, subtle brain changes might overlap with those observed in other neurodegenerative diseases, such as Alzheimer’s disease. Therefore, enhancing the accuracy and applicability of these MRI biomarkers will be a central focus in future research, particularly improving their capacity to differentiate between different neurodegenerative conditions.

We conducted mediation analyses again and found that, although CBF had a more significant modulation effect on the ALPS index compared to susceptibility, it remains unclear whether the mechanism of reduced cerebral blood flow is involved in the regulation of the lymphatic system. This may be related to the small sample size included in our study, which needs to be increased in future research to further explore the correlation between CBF and the ALPS index.

## 5 Limitation

Of course, there are several limitations in our study. First, we focused on the ALPS index values for the whole brain, but there may be regional differences in VaD. Future research should give more attention to specific regional variations. Second, this study only included VaD patients. Future research should include different groups of cognitive impairments caused by CSVD to explore the longitudinal changes in QSM, CBF, and ALPS index, in order to better understand the entire process of cognitive impairment progression. Third, we analyzed the differences between QSM and CBF using ROI-based methods. Future research should consider a whole-brain voxel-based approach for a more thorough analysis. Vascular risk factors such as hypertension, diabetes, and long-term medication use were not further stratified in this study. These factors could potentially affect cerebral blood flow, blood–brain barrier permeability, and lymphatic function, thereby interfering with changes in ALPS index, QSM, and ASL imaging parameters. Future studies with larger sample sizes should include and control for these variables to clarify their independent roles and interactions. Moreover, one of the limitations of this study is that we did not further explore the relationship between Fazekas score, WMH burden, and other imaging markers (such as ALPS index, QSM, etc.) or cognitive function. In this study, we chose the Fazekas score as an important diagnostic criterion for selecting VaD patients. Although the Fazekas score was a key basis for our inclusion criteria, it does not alone reflect the WMH burden, nor can it explore the relationship between WMH and other imaging markers (such as ALPS, QSM) or cognitive function in detail. Therefore, our study focused primarily on changes in imaging markers like ALPS, QSM, and ASL in VaD patients, without extending into detailed analyses of the relationship between WMH burden and these imaging markers and clinical outcomes. Future studies should expand the sample size and consider incorporating WMH burden and other imaging markers to further explore their relationships with CSVD and cognitive function.

## 6 Conclusion

To conclude, our findings suggest the complex pathological changes observed in the brains of VaD patients. These changes include dysfunction of the lymphatic system throughout the brain, iron deposition in occipital gyrus, decreased CBF in inferior frontal gyrus, rolandic operculum, insula, and heschl gyrus, all of which are associated with cognitive deficits. Moreover, decreased blood flow might play a role in controlling lymphatic system function, a discovery that needs more confirmation.

## Data Availability

The original contributions presented in the study are included in the article/supplementary material, further inquiries can be directed to the corresponding authors.

## Footnotes

1
https://fsl.fmrib.ox.ac.uk/

2
https://sepia-documentation.readthedocs.io/
